# Expression analysis of microRNA and lncRNA in visceral adipose tissue of obese and non-obese individuals

**DOI:** 10.18699/vjgb-25-48

**Published:** 2025-06

**Authors:** А. Бейркдар, Д.Е. Иванощук, О.В. Тузовская, Н.С. Широкова, Е.В. Каштанова, Я.В. Полонская, Ю.И. Рагино, Е.В. Шахтшнейдер

**Affiliations:** Федеральный исследовательский центр Институт цитологии и генетики Сибирского отделения Российской академии наук, Новосибирск, Россия Новосибирский национальный исследовательский государственный университет, Новосибирск, Россия; Федеральный исследовательский центр Институт цитологии и генетики Сибирского отделения Российской академии наук, Новосибирск, Россия Научно-исследовательский институт терапии и профилактической медицины – филиал Федерального исследовательского центра; Научно-исследовательский институт терапии и профилактической медицины – филиал Федерального исследовательского центра Институт цитологии и генетики Сибирского отделения Российской академии наук, Новосибирск, Россия; Федеральный исследовательский центр Институт цитологии и генетики Сибирского отделения Российской академии наук, Новосибирск, Россия; Научно-исследовательский институт терапии и профилактической медицины – филиал Федерального исследовательского центра Институт цитологии и генетики Сибирского отделения Российской академии наук, Новосибирск, Россия; Научно-исследовательский институт терапии и профилактической медицины – филиал Федерального исследовательского центра Институт цитологии и генетики Сибирского отделения Российской академии наук, Новосибирск, Россия; Научно-исследовательский институт терапии и профилактической медицины – филиал Федерального исследовательского центра Институт цитологии и генетики Сибирского отделения Российской академии наук, Новосибирск, Россия; Федеральный исследовательский центр Институт цитологии и генетики Сибирского отделения Российской академии наук, Новосибирск, Россия Научно-исследовательский институт терапии и профилактической медицины – филиал Федерального исследовательского центра Институт цитологии и генетики Сибирского отделения Российской академии наук, Новосибирск, Россия

**Keywords:** long non-coding RNA, microRNA, visceral adipose tissues, abdominal obesity, GAS5, SNHG9, miR-26A, miR-155, длинная некодирующая РНК, микроРНК, висцеральная жировая ткань, абдоминальное ожирение, GAS5, SNHG9, микроРНК-26A, микроРНК-155

## Abstract

Long non-coding RNAs (lncRNAs) and microRNAs (miRNAs) play important roles in all biological processes, including adipogenesis, lipid metabolism, and insulin response. Analyzing expression patterns of lncRNAs and miRNAs in human visceral fat tissue can enhance our understanding of their roles in metabolic disorders. Our research aims to investigate the expression of lncRNAs (ASMER1, SNHG9, P5549, P19461, and GAS5) and miRNAs (miR-26A, miR-222, miR-221, and miR-155) in visceral adipose tissues of individuals with abdominal obesity (n = 70) compared to their levels in non-obese participants (n = 31), using Real-Time PCR. Among the tested miRNAs, only miR-26A was significantly downregulated in the visceral adipose tissue of obese individuals, with no significant change in the expression of miR- 26A in obese people with or without type 2 diabetes. Similarly, of the tested lncRNAs, only GAS5 showed significantly higher expression levels in obese patients with type 2 diabetes (T2D) (n = 10) compared to obese patients without T2D (n = 60). To test possible interactions between the analyzed non-coding RNAs, we used Spearman’s bivariate correlation test. GAS5 expression levels showed a weak negative correlation (p < 0.05, rs = 0.25) with miR-155 levels in obese patients only. Conversely, a strong positive correlation (p <0.01, rs = 0.92) between SNHG9 and GAS5 was found in the non-obese group, with a weaker correlation in abdominally obese patients (p < 0.01, rs = 0.67); additionally, miR-26A and miR-155 levels were moderately correlated in the non-obese group (p < 0.05, rs = 0.47) and were found to correlate weakly in obese patients (p < 0.05, rs = 0.26). Our results showed that abdominally obese participants demonstrated higher expression levels of miR-26A in visceral adipose tissue and a significantly lower correlation between GAS5 and SNHG9 expression when compared to non-obese subjects.

## Abstract (ru)

Длинные некодирующие РНК (lncRNA) и микроРНК (miRNA) играют важную роль во всех биологических процессах, включая адипогенез, липидный обмен и инсулиновый ответ. Анализ экспрессии lncRNA и miRNA в висцеральной жировой ткани человека может способствовать более глубокому пониманию их роли в развитии метаболических нарушений. Исследование направлено на изучение уровней экспрессии lncRNA (ASMER1, SNHG9, P5549, P19461 и GAS5) и miRNA (miR-26A, miR-222, miR-221 и miR-155) в висцеральной жировой ткани у людей с абдоминальным ожирением (n = 70) по сравнению с уровнями их экспрессии у лиц без абдо- минального ожирения (n = 31) методом количественной ПЦР в реальном времени. Среди протестированных miRNA уровень экспрессии miR-26A был снижен в висцеральной жировой ткани у людей с ожирением. Среди изученных lncRNA GAS5 показал значительное повышение уровня экспрессии у пациентов с ожирением и са- харным диабетом 2-го типа (СД2) (n = 10) по сравнению с лицами с ожирением без СД2 (n = 60). Уровни экс- прессии GAS5 показали слабую отрицательную корреляцию (p < 0.05, rs = 0.25) с уровнями miR-155 только у пациентов с ожирением. Положительная корреляция (p < 0.01, rs = 0.92) между SNHG9 и GAS5 была обнаружена в группе людей без ожирения, с уменьшением коэффициента корреляции у пациентов с ожирением (p < 0.01, rs = 0.67). Кроме того, уровни miR- 26A и miR-155 умеренно коррелировали в группе без ожирения (p < 0.05, rs = 0.47) и демонстрировали слабую корреляцию у пациентов с ожирением (p < 0.05, rs = 0.26). Наши резуль- таты показали, что у пациентов с абдоминальным ожирением наблюдаются повышенные уровни экспрессии miR- 26A в висцеральной жировой ткани и слабая корреляция между экспрессией GAS5 и SNHG9 по сравнению с лицами без абдоминального ожирения.

## Introduction

The prevalence of obesity has been rising over the last four decades and it is currently considered a global pandemic (Valenzuela et al., 2023). Visceral fat accumulation has a stronger association with metabolic disorders compared to subcutaneous fat, despite both primarily consisting of white adipose tissue (Nicklas et al., 2003).

The complexity of white adipose tissue lies in its complex role as an endocrine organ, secreting various hormones, enzymes, growth factors, and multiple non-coding RNAs, all of which regulate numerous metabolic processes in the organism (Lustig et al., 2022). In the last decade, studies have been focusing on long non-coding RNAs (lncRNAs) and microRNAs (miRNAs) as key regulators in the fine-tuning of gene function across various biological processes (Mohanty et al., 2015; Rupaimoole, Slack, 2017). lncRNAs can be transcribed from different regions in the DNA and are typically categorized into three types: intergenic, antisense, and intronic. According to the latest recommendation, lncRNAs are transcripts longer than 500 nucleotides (Mattick et al., 2023), are less evolutionarily conserved than mRNAs, contain fewer exons, are characterized by lower splicing efficiency than mRNAs. lncRNAs are less abundantly expressed than other non-coding RNAs (Statello et al., 2021). Their functions include RNA-chromatin, RNA-RNA, and RNA-protein interactions (Ferrer, Dimitrova, 2024). The functioning mechanism of lncRNA involves the regulation of chromatin structure and transcription, usually through the methylation of enhancer regions (Li W. et al., 2016). lncRNAs interact with mRNAs, modifying their splicing and stability (Morrissy et al., 2011). Additionally, lncRNA can hybridize with mature RNAs at the 5ʹ-region, increasing its translation efficiency (Carrieri et al., 2012; Zucchelli et al., 2015).

Altered expression profiles of lncRNAs have been found in patients with acute myocardial infarction (Zhong et al., 2018), multiple malignancies (Li L. et al., 2021), and, most recently, Alzheimer’s disease (Zhang M. et al., 2019; Zhang J.-J. et al., 2021). Studies associating lncRNAs with metabolic disorders have investigated their roles in adipose tissue regulation and development (Tello-Flores et al., 2021; Corral et al., 2022; Sufianov et al., 2023), with accumulating evidence emphasizing their roles in obesity, type 2 diabetes, and other related disorders (Liu et al., 2018; Su et al., 2023).

miRNAs are short in length, approximately 22 nucleotides long, usually derived from longer primary miRNA (premiRNA) transcripts. Most miRNAs are processed from a noncoding transcript originating from the intron or non-coding regions, and occasionally from exons of protein-coding genes (Dexheimer, Cochella, 2020).

The direct association of microRNAs with a wide variety of diseases – from various malignancies (Reddy, 2015; Ali Syeda et al., 2020) and autoimmune diseases (Zhang L. et al., 2020), to metabolic disorders including obesity (Veie et al., 2023) and type 2 diabetes (Miao et al., 2018) – is well-established. Currently, miRNAs are being explored as biomarkers in obesity (Gouda et al., 2023) and as therapeutic targets due to their short size and promising in vitro (Acharya et al., 2019) and in vivo results (Lhamyani et al., 2021).

miR-155, miR-221, and miR-222 were found to be decreased during adipogenic differentiation of human mesenchymal stromal cells, suggesting their role as negative regulators of the process (Skårn et al., 2012). Moreover, miR-26A overexpression in transgenic mice led to reduced visceral fat levels and an improved lipid profile (Zeng H. et al., 2021). miR- 222 blood levels were higher in obese subjects after surgeries leading to weight loss, while in the same subjects, miR-221 levels were reduced (Ortega et al., 2013). Circulating levels of miR-155 were higher in obese patients, while miR- 26A was downregulated in the same group (Kim et al., 2020).

Non-coding RNAs function as networks that, if disrupted, could lead to multiple disorders including, but not limited to, obesity (Ma et al., 2023); miR-26A is found to be downregulated by the lncRNA GAS5 sponge mechanism in degenerated nucleus pulposus cells (Tan et al., 2021). Additionally, GAS5 is suggested to participate in the pathogenesis of pneumonia by downregulating miR-155 and inhibiting apoptosis (Wang et al., 2021). The miRNA-lncRNA interaction network in obesity has recently gained interest. Guo and Cao (2019) described a potential interaction of lncRNA RP11-552F3.9 with miR- 130b and miR-23a in LEP gene regulation and adipogenic differentiation. Additionally, RP11-142A22.4 was identified as a functional site for binding miR-587, making it unavailable and subsequently promoting adipogenesis (Zhang T. et al., 2020). Another example is lncRNA Adi, discovered in 2020, which was found to bind to miR-449a in adipose-derived stem cells, inducing their adipogenic differentiation (Chen et al., 2020).

Previous studies investigating the expression profiles of lncRNAs in obese patients were limited to describing blood levels (Sun et al., 2016; Cabiati et al., 2022; Rasaei et al., 2024) and/or had limited population samples (Tait et al., 2020; Tan et al., 2021). Additionally, in 2016, Sun et al. identified new lncRNAs (P19641, P5549, P21015) that are correlated with obesity, but fell short of identifying the splicing variants expressed in blood samples (Sun et al., 2016). Similarly, Lv et al.’s study describing GAS5 function in visceral fat tissues included only female subjects (Lv et al., 2022).

Despite the attempt by Kim et al. to identify differentially expressed miRNAs in visceral fat tissues, their population consisted only of females and was limited to 20 subjects (Kim et al., 2020). The same limitation was noted in the study by Capobianco et al. (2012).

Due to the limitations of the aforementioned studies, our research aims to first identify the specific splicing variant of P19641, P5549, SNHG9, and ASMER1 in human visceral fat tissue samples. Subsequently, we aim to measure the expression levels of multiple lncRNAs (P19641, P5549, SNHG9, ASMER1, and GAS5) and miRNAs (miR-221, miR-222, miR-155, and miR-26A) in our population.

## Materials and methods

Study design. The study protocol was approved by the local Ethics Committee of the Institute of Internal and Preventive Medicine – Branch of the Institute of Cytology and Genetics, Siberian Branch of the Russian Academy of Sciences, Novosibirsk, Russia (Protocol No. 66 from October 10, 2023) – and was conducted in accordance with the principles of the Declaration of Helsinki of the World Medical Association. Informed consent was obtained from all participants

A total of 101 accepted individuals underwent a comprehensive clinical examination program. This included the collection of sociodemographic data, administration of a standardized questionnaire on smoking and alcohol use, documentation of chronic disease history, and recording of medication usage; the main medication used is described in Table 1, additional information about administered medications can be found in the supplementary file (Supplementary Material 1)1. Additionally, the program involved the Rose cardiological questionnaire, anthropometric measurements (height, body weight, and waist circumference), and blood pressure measurements. Blood serum samples were collected for biochemical assays measuring total cholesterol, high-density lipoprotein cholesterol (HDL-C), low-density lipoprotein cholesterol (LDL-C), triglycerides, and fasting glucose levels.

**Table 1.**
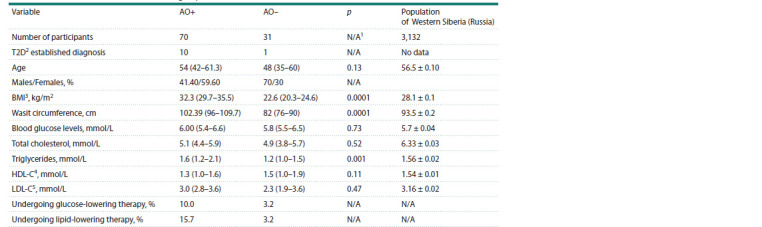
Clinical characteristics, body composition, and biochemical variables of the study individuals divided into an obese (AO+) and a non-obese group (AO–) Note. Values are indicated as median and (25–75 % IQR), while reference values are indicated as mean ± standard error of the mean. 1 Not applicable, 2 type 2 diabetes, 3 body mass index, 4 high-density lipoprotein cholesterol, 5 low-density lipoprotein cholesterol.


Supplementary Materials are available in the online version of the paper: https://vavilovj-icg.ru/download/pict-2025-29/appx15.pdf


Lipid levels (cholesterol, triglycerides, and low-density and high-density lipoprotein cholesterols) and glucose concentration were measured using a biochemical analyzer KoneLab 300i (Thermo Fisher Scientific Oy, Finland) with Thermo Fisher Scientific reagents (USA). The values of LDL- C concentration were calculated using the Friedwald formula. The atherogenic coefficient was calculated using the formula: IA = (TC − HDLC)/HDLC. The levels of leptin and adiponectin were determined by multiplex analysis using the Human Adipokine Magnetic Bead Panel 1 kit (EMD Millipore Corporation, Darmstadt, Germany) on a Luminex 20 MAGPIX flow cytometer (Luminex Corporation, Austin, TX, USA).

All 101 subjects scheduled for elective surgery were included in the study based on the main inclusion criteria of having no records of chronic inflammation or symptoms of acute inflammation prior to surgery. The participants were subsequently divided into two groups: 70 participants with abdominal obesity (AO+) and 31 non-obese subjects (AO–) (Table 1). Abdominal obesity was defined as a body mass index (BMI, kg/m2) of ≥25.0 or a waist circumference (WC) of ≥80 cm in women and ≥94 cm in men, in accordance with the latest medical guidelines (Yumuk et al., 2015; Dedov et al., 2021). Type 2 diabetes (T2D) was diagnosed based on the criteria set forth by the American Diabetes Association. No adjustment for age was applied since the study group was intended to represent only the population aged 45–55. Additionally, no adjustment for sex or medications was applied because the resulting subgroups would be relatively small and could generate statistically unreliable data (Table 1). Individuals exhibiting chronic or acute inflammation on the day of surgery, as well as pregnant women and women on maternity leave, were excluded from the study.

Sample collection and RNA extraction. During the operation, samples of visceral adipose tissue were collected, washed with PBS and subsequently preserved in RNAlater solution (ThermoFisher, USA) at –20 °C until further handling. lncRNA was extracted using the total RNA extraction protocol, microRNA was extracted using the microRNA extraction protocol. Both procedures were performed with the Total and microRNA Extraction Kit (LRU-100-50) (Biolabmix, Russia). All samples were treated with RNase-free DNase (Biolabs, USA).

The concentration of yielded RNA was determined using the BioTek Epoch Analyzer (Agilent Technologies, USA), and the quality was estimated by the absorbance ratios A260/230 and A260/280. Additionally, the quality of RNA preservation was assessed by determining the integrity of 28S, 18S, and 5S bands on 1 % agarose gel electrophoresis

lncRNA reverse transcription and relative quantification. Reverse transcription of total RNA was performed using the MMuLV Reverse Transcription Kit. A total of 1,000 ng of each sample was used in a 40 μL reaction volume, which included Random Primer6, oligo(dT), and 5X reaction buffer (Kolenda et al., 2021). All cDNA samples were diluted to a total volume of 100 μL.

For P5549, P19641, ASMER1, and SNHG9 lncRNAs, we designed a multiple set of specific primers for each splicing variant listed in the Ensembl database (Ensembl.org) at the start of the study (October 2023). For GAS5 lncRNA, we used a single set of primers specific for the canonical transcript ENST00000702964.1. All primers were designed using the PrimerBlast tool (NCBI, USA). Primer efficiency was determined by the standard curve method with four crossing points. Specificity was assessed by analyzing melting curves from 65 to 95 °C with a 0.5 °C increment per cycle and by determining product length using 5 % acrylamide gel. Conventional PCR for splicing variant identification and qPCR for relative expression analysis were performed using SYBR GREEN I intercalating dye master mix “BioMaster HS-qPCR SYBR Blue (2×)” (Biolabmix, Russia) on the LightCycler 96 system (Roche, Switzerland). The amplification program was 95 °C for 3 min, followed by a 3-step amplification (95 °C for 15 sec, 63 °C for 15 sec, 72 °C for 20 sec) for a total of 40 cycles, followed by product melting as described above. Sequences of all primers used and standard curves are available in the Supplementary Material 1. Expression of all lncRNAs was normalized to the stable reference gene GAPDH expression levels (Mehta et al., 2010; Ebrahimi et al., 2020).

miRNA reverse transcription and relative quantification. Reverse transcription of microRNA was performed using the stem-loop pulsed reverse transcription protocol with 150 μL of the extracted microRNA (Varkonyi-Gasic et al., 2007). Expression was measured using SYBR GREEN I intercalating dye master mix “BioMaster HS-qPCR SYBR Blue (2×)” (Biolabmix, Russia) on the LightCycler 96 system (Roche, Switzerland). Primers designed for the reverse transcription product included a reverse primer specific for the stem-loop structure and a forward primer specific for the miRNA of interest (Varkonyi-Gasic et al., 2007). Efficiency was determined by the standard curve method with four crossing points. Specificity was assessed by analyzing melting curves from 65 to 95 °C with a 0.5 °C increment per cycle and by determining product length using 5 % acrylamide gel analysis. For miRNA quantification, we designed reverse transcription primers and specific qPCR primers using the online tool sRNAPrimerDB (srnaprimerdb.com). Sequences of all primers used and standard curves are available in the Supplementary Material 1. Expression of all microRNAs was normalized to miRNA-103, given its proven stable expression levels in visceral fat tissue (Ragni et al., 2021).

lncRNA sequence verification by Sanger sequencing. Sanger sequencing was used to verify the PCR amplification product sequence. The PCR product was purified using the Reaction Mixtures DNA Isolation Kit (DR-50, Biolabmix, Russia). Sequencing was performed using the BigDye Terminator v3.1 Kit (ThermoFisher, USA) according to the manufacturer’s protocol, and capillary electrophoresis was conducted using the SeqStudio Genetic Analyzer (ThermoFisher, USA). All sequences were verified using the Blastn online alignment tool (Ensembl.org). Alignment options were set to default, and only the first sequence hit was reported (Table 2) with an identity of ~100 % and the highest percentage of coverage.

**Table 2.**
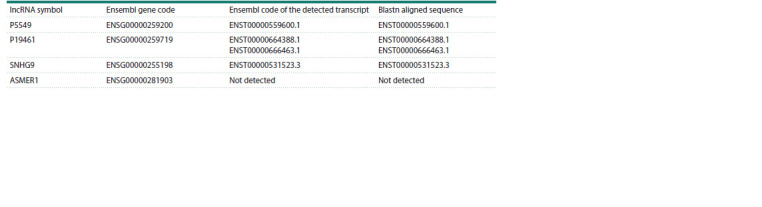
Splicing variants of the investigated lncRNAs identified to be expressed in visceral fat tissue

Statistical analysis. The ΔCt method was used to calculate the relative expression (2–ΔCt, fold of expression relative to the reference gene by the formula ΔCt = (Ctmeasured transcript – – Ctreference transcript) of lncRNA and microRNA (Livak, Schmittgen, 2001). All variables were tested for normal distribution using the Kolmogorov–Smirnov test. The Mann–Whitney test was used for group comparison. A p-value of 0.05 was considered significant (*), and p < 0.01 was considered highly significant (**). No cutoff for the fold change was assigned. Variables are presented as the median and 25–75 % interquartile range (IQR). Correlation between continuous variables and expression values of both miRNA and lncRNA was tested using Spearmans’s bivariate correlation analysis and results were reported as Spearman’s rank correlation coefficient and p-value for statistical significance. All statistical analyses were performed using SPSS software version 26 for Windows (IBM, USA). All graphs were generated using Prism GraphPad software version 8.2.1 for Windows (Dotmatics, USA).

## Results

Subject characteristics

Clinical characteristics of the studied groups are presented in Table 1. A comparative analysis of obese and non-obese individuals revealed differences in triglyceride levels ( p = 0.01). Values of reference biochemical markers for the West Siberian population were described in a survey conducted in 2019 (Semaev et al., 2019).

We aimed, first, to identify the transcribed splicing variant of all investigated lncRNAs. Second, we quantified various lncRNAs and miRNAs for subsequent statistical analysis of correlation with phenotypes of obesity, diabetes, and different biochemical markers. The final section investigates the possible correlation between lncRNA and miRNA expression, which might indicate potential interaction or a common regulatory pathway involved in adipogenesis and the pathology of obesity.

lncRNA expression

Out of the two explored alternative expression forms of lncRNA P5549, we identified ENST00000559600.1 to be solely expressed in our sample pool. ENST00000559600.1 is defined to be the canonical variant in the Ensembl database, has exons 2, 3 and 4 alternatively spliced, while exons 6, 7 and 8 are kept (Table 2). On the other hand, P19461 was found to be expressed in two alternative forms out of the five forms that we tested for. ENST00000664388.1 is the only transcript with exon 10 kept and defined as the canonical form by the Ensembl database. ENST00000666463.1 is the shortest splicing form of P19461 with only one exon (exon 1). We also tested for the expression of two different transcripts of SNHG9 and found the canonical transcript ENST00000531523.3 to be the only expressed form in visceral fat samples; this transcript has exon 2 out of all three exons alternatively spliced. Finally, none of the four investigated ASMER1 splicing variants had detected expression levels (Table 2). Sequence data of all PCR products used for identification of lncRNAs are available in the Supplementary Material 1.

**Fig. 1.**
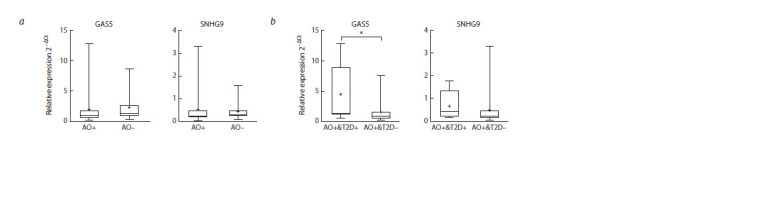
A box plot representing the relative expression of lncRNA GAS5 and SNHG9. a, Comparison of lncRNA expression in the obese group (AO+) and the non-obese group (AO–); b, comparison of lncRNA expression between obese subjects diagnosed with type 2 diabetes (AO+&T2D+) and obese subjects without an established diagnosis of T2D (AO+&T2D–). The box represents the 25th–75th percentile range, error bars represent the minimum and maximum values, the median is represented as a line inside the box, and the mean, as a + sign. * p < 0.05.

The fluorescence signal of specific PCR products for lncRNA P5549 and P19461 was detected at late cycles and was lower than the detection limits of LightCycler 96, which made further precise relative quantification not possible by a simple qPCR method. Relative quantification showed no significant difference of mean SNHG9 and GAS5 expression levels between obese patients and non-obese subjects. After adjusting for type 2 diabetes diagnosis, GAS5 expression levels were significantly higher (~3 fold of change of the mean) ( p = 0.03) in obese subjects with T2D compared to obese patients without T2D (Fig. 1). Expression levels of measured lncRNAs are shown in Table 3.

**Table 3.**
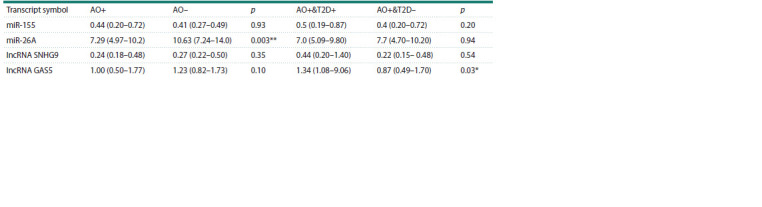
Expression levels of different measured lncRNAs and miRNAs in visceral fat tissues of obese patients (AO+) and non-obese participants (AO–), as well as in obese individuals with abdominal obesity and type 2 diabetes (AO+&T2D+) and obese participants without type 2 diabetes (AO+&T2D–)

Correlation analysis between lncRNA expression levels and various metabolic parameters, such as BMI, waist circumference, total cholesterol, and lipid profile, did not show any significant correlation in either of the studied groups. The results of correlation tests performed on lncRNAs are provided in the Supplementary Material 2.

microRNA expression

**Fig. 2.**
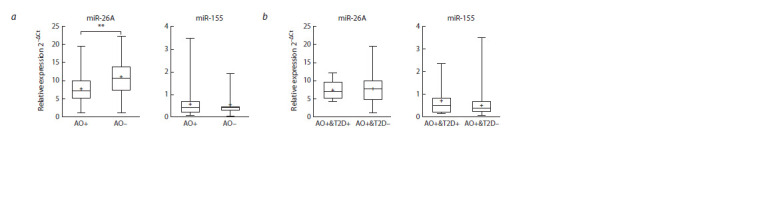
A box plot representing the relative expression of miR-26A and miR-155. a, Comparison of miRNAs expression in the obese group (AO+) and the non-obese group (AO–); b, comparison of miRNAs expression between obese subjects diagnosed with type 2 diabetes (AO+&T2D+) and obese subjects without an established diagnosis of T2D (AO+&T2D–). The box represents the 25th–75th percentile range, error bars represent the minimum and maximum values, the median is represented as a line inside the box, and the mean, as a + sign. ** p <0.01.

Expression levels of miR-221 and miR-222 were undetectable using the method described before. Expression levels of miR- 155 were not significantly changed between the obese and the non-obese group, while expression levels of miR- 26A were significantly lower in the obese group (~1.5 fold of change of the mean) ( p < 0.01) (Fig. 2). After adjustment for type 2 diabetes diagnosis, miR-155 and miR-26A expression levels showed no significant differences between obese patients diagnosed with T2D compared to obese individuals without T2D (Fig. 2). Mean expression levels of miRNAs in the studied groups are described in Table 3.

We also performed a correlation test between miR-26A and miR-155 expression levels and multiple metabolic parameters. In obese subjects, miR-155 expression showed a moderate positive correlation with BMI (rs = 0.32, p = 0.009). Interestingly, in non-obese subjects, the opposite pattern of correlation was observed, where miR-155 negatively correlated with both BMI (rs = –0.44, p = 0.029) and waist circumference (rs = –0.44, p = 0.027). A table of all correlation tests performed on miRNAs is provided in the Supplementary Material 2.

Correlation of lncRNA-lncRNA, miRNA-miRNA and lncRNA-miRNA expression levels

We tested the correlation between miRNA and lncRNA expression levels (Table 4). In obese patients, lncRNA GAS5 showed a weak negative correlation with miR-155 expression levels (rs = 0.25, p < 0.05), whereas this correlation was not observed in non-obese participants. Additionally, no correlation was found between GAS5 and miR-26A expression levels in either group. Similarly, SNHG9 did not correlate with any of the investigated miRNAs in either group. Interestingly, the non-obese group showed a very strong correlation (rs = 0.92, p <0.0001) between SNHG9 and GAS5 expression levels. However, the correlation between SNHG9 and GAS5 expression levels in obese subjects was moderate and significant (rs = 0.67, p < 0.0001). Similarly, miR-26A and miR-155 displayed a moderate but significant (rs= 0.47, p < 0.05) correlation in the non-obese group; the same correlation was reduced and manifested to be weak in obese participants (rs = 0.26, p < 0.05). Scatter plots representing bivariate correlations are available in the Supplementary Material 2.

**Table 4.**
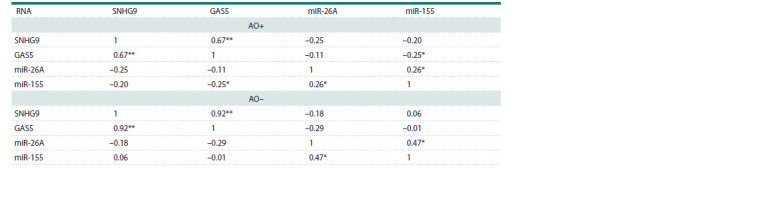
Spearman’s correlation between pairs of measured non-coding RNAs in the obese (AO+) and the non-obese group (AO–) Note. The strength of correlation is described by the Spearman’s rank correlation coefficient (rs). * Indicates a significant correlation with 2-tailed p-value < 0.05, ** indicates a significant correlation with 2-tailed p-value < 0.01

## Discussion

The first objective of our study was to identify the specific transcription variant expressed in visceral fat tissue for each of the lncRNAs P5549, SNHG9, P19641, and ASMER1 (Table 2). Using specific primers and Sanger sequencing, we revealed for the first time that lncRNA P5549 was represented by the transcript ENST00000559600.1, and SNHG9 was solely identified as ENST00000531523.3. P19641 was expressed in two different splicing forms: ENST00000664388.1 and ENST00000666463.1, which can be attributed to the inefficient splicing of some lncRNAs (Li L. et al., 2021). The specific splicing variant expressed in visceral adipose tissue suggests a functional significance for the specifically transcribed exons in the interaction of the studied lncRNA with its targets, whether they be DNA, mRNA, or microRNA. Further studies are necessary to identify the exact functioning sequences of the identified lncRNAs, which could facilitate the implementation of these sequences as future therapeutic targets. Additionally, no expression of any of the tested variants for ASMER1 lncRNA was detected. This may be a sign of very low expression levels that are undetectable by the method used for investigation or an indication of low RNA stability.

Next, we aimed to compare the expression levels of lncRNAs SNHG9 and GAS5 between our obese and nonobese groups. Our analysis showed no significant difference in the mean expression levels of either of the analyzed lncRNAs between the two groups. However, GAS5 expression was significantly higher in obese subjects with type 2 diabetes (T2D) compared to obese subjects without T2D (Fig. 1). This contradicts the results from multiple studies showing lower GAS5 expression levels in blood samples collected from T2D patients compared to non-obese subjects (Fawzy et al., 2020; Luo et al., 2020; Ahmadi et al., 2024). GAS5 was found to be induced by proinflammatory mediators (Mameli et al., 2016), which might explain its higher levels in obese patients with T2D, a condition always linked to chronic adipose tissue inflammation (Zatterale et al., 2020). Our results suggest a different expression pattern of GAS5 in visceral fat tissues and might reflect the delicate balance between its function as an adipogenesis inhibitor and a proinflammatory factor. Despite the novelty of our finding, further verification in a larger population is required, in addition to investigating the mechanisms by which GAS5 might regulate fat metabolism

The expression levels of miR-221 and miR-222 were undetectable in our study, which might be due to tissue-specific expression patterns or methodological differences compared to previous studies (Markovic et al., 2020; Chan et al., 2022). miR-26A was significantly downregulated in obese subjects, whereas miR-155 showed no significant dysregulation between the two groups (Fig. 2). The downregulation of miR- 26A in obesity aligns with the findings of Kim et al. (2020) and emphasizes the role of miR-26A in adipogenesis and metabolic regulation described in previous studies (Acharya et al., 2019; Zeng H. et al., 2021). Additionally, miR- 155 expression was correlated with current studies that are examining the potential of non-coding RNAs as biomarkers and therapeutic targets (Winkle et al., 2021). GAS5 has been presented as a promising biomarker in breast cancer treatment and prognosis (Grossi et al., 2023). Meanwhile, miR-26A has already been tested as a therapeutic molecule attenuating visceral fat accumulation in a mouse model (Zeng H. et al., 2021). Our results highlight the necessity for novel research investigating GAS5 as a therapeutic target in metabolic disorders. Additionally, we emphasize the need for further evidence of the efficacy of miR-26A as a potential therapeutic molecule in future preclinical and clinical studies.

Our study also explored potential interactions between the studied non-coding RNAs. We found no correlation between SNHG9 expression and miR-26A or miR-155. On the other hand, GAS5 showed a weak negative correlation with miR- 155 levels in visceral fat tissue samples of obese individuals; we didn’t observe a similar correlation in nonobese subjects (Table 4). Previous studies described a negative correlation between GAS5 and miR-155-5p in regulatory pathways related to inflammation and apoptosis. GAS5 was described to sponge and regulate miR-155, leading to the upregulation of SIRT1 and suppression of the inflammatory response to lipopolysaccharides (Zeng Z. et al., 2023). Additionally, GAS5 and miR-155 were inversely correlated in pneumonia patients, where GAS5 was downregulated and miR-155 was upregulated in plasma samples. GAS5 overexpression decreased miR-155 expression in human bronchial epithelial cells (Wang et al., 2021). This finding requires further confirmation in experimental studies, which may reveal a similar mechanism of regulation in adipose tissue as in inflammatory responses.

GAS5 expression levels showed no correlation with miR- 26A in either of the studied groups. This finding does not align with the results of Tan et al. (2021) where GAS5 was responsible for the downregulation of miR-26A. Therefore, additional studies exploring miR-26A interaction network in visceral adipose tissue are required to clarify the exact mechanism by which miR-26A is negatively regulated in obese individuals.

In an attempt to expand our knowledge of the intricate network of non-coding RNAs, we also investigated the correlation between SNHG9 and GAS5 expression levels. Interestingly, we found a very strong, almost linear, correlation between the two lncRNAs in non-obese subjects (rs = 0.92). This correlation was significantly reduced in obese patients (rs = 0.67). Previous studies investigating expression levels of SNHG9 and GAS5 showed inversed correlation in clear cell renal cell carcinoma (Yang et al., 2020) and glioblastoma (Ji et al., 2020), but no common regulation mechanism was suggested. To the best of our knowledge, such correlation between SNHG9 and GAS5 has never been described before in studies of metabolic disorders. Hence, we can only suggest the existence of coordinated regulation between these two lncRNAs in normal metabolic conditions. This coordination could be disrupted in the condition of obesity, leading to a weaker correlation in the expression levels of the two lncRNAs. However, further in vitro and in vivo experiments are required to confirm any type of common regulation mechanism

A moderate correlation between miR-26A and miR-155 (rs = 0.47) was found in the non-obese group, and this correlation was shown to be weak in obese patients (rs = 0.26), which partially aligns with the correlation pattern of the above-described lncRNAs. Previous studies showed parallel downregulation of miR-26A and miR-155 in subjects with obesity (Kim et al., 2020) and multiple sclerosis (Mameli et al., 2016), but none of the studies attempted to test for a correlation between the expression levels of the two miRNAs. Such a correlation in expression levels might be evidence of a common regulation mechanism for miR-26A and miR-155. The reduced correlation in obese patients in our results aligns with the previous observation in lncRNA interaction that suggests the existence of a common regulation mechanism for both miRNAs, which might be disrupted in the case of obesity.

Our study included 101 participants divided into 70 obese patients and 31 non-obese individuals, all within the age group of 45–55 years. The study group was not adjusted for sex and types of medications, which is the first of several limitations we acknowledge. However, previous data investigating the expression of different non-coding RNAs in visceral fat tissue are extremely rare, and our chosen targets had not been investigated in this tissue before. Additionally, we aimed to generate first-time data that can prompt further investigation in this direction.

The limitations of our study also include the use of conventional PCR and subsequent Sanger sequencing for the identification of lncRNA splicing variants. While this method is highly specific, it is limited due to the small range of transcripts it can identify. We recommend further studies using transcriptomic methods to identify novel lncRNAs and new forms of expression for existing RNAs. Another limitation of our study was the inability to quantify the scarcely expressed lncRNAs, which might require preamplification or the use of digital PCR. Finally, our study describes correlations and statistical differences that require further confirmation or rejection by experimental in vitro and in vivo studies. Transcriptomics might also aid in identifying and quantifying various novel transcripts of lncRNAs expressed in visceral fat tissue, deepening our understanding of adipogenesis and fat metabolism.

## Conclusion

In conclusion, the precise network of non-coding RNAs is once more shown to be associated with the development of metabolic and various other diseases. Our results indicated a different pattern of microRNA and lncRNA expression in individuals suffering from obesity and T2D. This dissimilarity highlights the important role of the investigated non-coding RNAs in the formation and differentiation of visceral adipose tissue. Additionally, our results displayed a specific miRNAmiRNA and lncRNA-lncRNA correlation pattern in non-obese individuals, which requires further investigation. Understanding these associations can lead to building a better map of the interaction network in the absence of metabolic disorders. This map can serve as a reference for understanding all possible abnormal or alternative pathways regulating the intricate networks in cases of obesity and other metabolic disorders.

## Conflict of interest

The authors declare no conflict of interest.
